# Association between psychosocial consequences of visual dysfunction and depressive symptom burden in multiple sclerosis and neuromyelitis optica spectrum disorder

**DOI:** 10.3389/fneur.2026.1975600

**Published:** 2026-10-20

**Authors:** Guoxun Zhang, Han Wang, Xiaojia Sun, Yu Liu, Chunyang Li, Xiao Yang, Jia Guo, Huaxing Meng, Xuelan Ye, Di Zhong, Hongmei Wang, Yanru He, Dongjie Sun, Jinting Zhu, Lili Ge, Qiwu Zhou, Kin Chiu, Runze Zhao

**Affiliations:** 1 Department of Neurology, Xianyang Hospital of Yan'an University, Xianyang, China; 2 Department of Ophthalmology, Eye Institute of Chinese PLA, Xijing Hospital, Fourth Military Medical University, Xi’an, China; 3 Department of Neurology, General Hospital of Ningxia Medical University, Yinchuan, China; 4 Department of Neurology, The Second Hospital of Lanzhou University, Lanzhou, China; 5 Department of Neurology, The First Affiliated Hospital of Shanxi Medical University, Taiyuan, China; 6 Department of Neurology, Qinghai University Affiliated Hospital, Xining, China; 7 Department of Neurology, The First Affiliated Hospital of Harbin Medical University, Harbin, China; 8 Department of Psychology, The University of Hong Kong, Pokfulam, Hong Kong SAR, China

**Keywords:** depression, multiple sclerosis, Neuromyelitis optica spectrum disorder, patient-reported outcomes, psychosocial functioning, vision-related quality of life, visual impairment

## Abstract

Visual dysfunction and depressive symptoms are common and often disabling manifestations of multiple sclerosis (MS) and neuromyelitis optica spectrum disorder (NMOSD). In this multicenter cross-sectional study, 458 adults with MS (*n* = 370) or NMOSD (*n* = 88) were recruited from six tertiary hospitals in China. Vision-related quality of life (VRQoL) was assessed using the National Eye Institute Visual Function Questionnaire-25 (NEI VFQ-25), depressive symptoms using the Hospital Anxiety and Depression Scale depression subscale (HADS-D), and objective visual impairment using World Health Organization blindness criteria. Lower NEI VFQ-25 scores were associated with HADS-D screen positivity after adjustment for measured covariates (OR 2.33 per 10-point decrease, 95% CI 1.81–3.00; *p* < 0.001), whereas WHO-defined blindness was not significantly associated with HADS-D screen positivity in the fully adjusted model. In domain-specific analyses, associations for Core Visual Function were attenuated after mutual adjustment, whereas Psychosocial scores remained associated with HADS-D screen positivity. These cross-sectional findings suggest that vision-related patient-reported outcomes capture aspects of functional and psychosocial disease burden that are not fully reflected by a binary classification of visual impairment.

## 1 Introduction

Visual dysfunction is a common and disabling manifestation of multiple sclerosis (MS) and neuromyelitis optica spectrum disorder (NMOSD). Damage to the optic nerve and visual pathways frequently results in impaired visual function, reduced mobility, difficulties with daily activities, and diminished quality of life (1–3). Depressive symptoms are also highly prevalent in both disorders and contribute substantially to disability, reduced social participation, and poorer health outcomes (4–6). Although visual impairment and depression frequently coexist in inflammatory demyelinating diseases, the relationship between these dimensions of disease burden remains incompletely understood.

Visual dysfunction is traditionally assessed using objective measures such as visual acuity, visual field testing, and ophthalmological examination. While these measures are essential for quantifying visual impairment, they may not fully reflect its impact on daily functioning and well-being. Patients with similar levels of objective visual impairment often report markedly different degrees of functional limitation, emotional distress, and social participation (7, 8). Consequently, patient-reported outcomes are increasingly recognized as complementary measures of disease burden in neuroimmunological disorders (9, 10). Recent cross-sectional evidence from Ecuador similarly found that quality of life was associated with anxiety, depression, fatigue, and disability among people with MS and NMOSD, with greater quality-of-life impairment observed in the NMOSD group (11).

The National Eye Institute Visual Function Questionnaire-25 (NEI VFQ-25) is one of the most widely used instruments for evaluating vision-related quality of life (VRQoL) in ophthalmic and neurological diseases (12). In addition to visual functioning, it assesses the impact of visual difficulties on independence, social functioning, role performance, and emotional well-being (13). These domains may be particularly relevant in MS and NMOSD, where visual dysfunction frequently coexists with physical disability, fatigue, cognitive impairment, and psychological distress (14, 15).

Previous studies have reported associations between VRQoL and mood-related outcomes (16, 17). In NMOSD, a recent cross-sectional mediation analysis further linked anxiety and depression with poorer quality of life and suggested that several neurological symptom clusters may account for part of these associations (18). However, it remains unclear whether these associations are independent of objective visual impairment and whether vision-related patient-reported outcomes provide information beyond conventional measures such as World Health Organization (WHO)-defined blindness. Clarifying this relationship may improve understanding of the broader impact of visual dysfunction and help identify patients whose perceived disease burden is not fully reflected by objective visual findings.

Therefore, in this multicenter cross-sectional study, we examined the association between vision-related quality of life and depressive symptom burden in adults with MS and NMOSD. We investigated whether this association persisted after adjustment for severe visual impairment and explored the associations of conceptually defined visual-function and psychosocial domains of the NEI VFQ-25 with depressive symptom burden. We hypothesized that both components of vision-related quality of life would be associated with depressive symptom burden and examined their estimates within the same analytical framework.

## 2 Materials and methods

### 2.1 Study design and participants

This multicenter cross-sectional study was conducted between October 2024 and March 2025 at six tertiary hospitals in China: Yan’an University Xianyang Hospital, Xijing Hospital, General Hospital of Ningxia Medical University, The Second Hospital of Lanzhou University, The First Affiliated Hospital of Harbin Medical University, and Affiliated Hospital of Qinghai University. Consecutive adult patients were recruited during routine outpatient and inpatient visits to participating neurology and ophthalmology services. Because this was a multicenter observational study, no formal prospective sample-size calculation was performed; the analytic sample comprised consecutively eligible participants recruited during the study period.

Eligible participants were aged ≥18 years and had a confirmed diagnosis of MS according to the 2017 McDonald criteria (19) or NMOSD according to the 2015 International Consensus Diagnostic Criteria (20). Exclusion criteria included acute optic neuritis within 3 months of assessment, ocular conditions unrelated to MS or NMOSD that could affect visual function, a history of major psychiatric disorders predating neurological diagnosis, or current treatment with antidepressant or anxiolytic medications. A total of 11 potentially eligible patients were excluded because of current antidepressant or anxiolytic treatment. The medication-related exclusion was intended to reduce potential effects of psychotropic treatment on self-reported symptoms; it may, however, limit generalizability by underrepresenting patients receiving treatment for depression or anxiety.

### 2.2 Clinical and visual assessments

Demographic variables included age, sex, educational attainment, and marital status. Disease-related variables included disease subtype, disease duration, first ocular episode, and history of ocular involvement.

Objective visual impairment was classified according to WHO criteria for blindness. Participants were classified using ophthalmological examinations performed on the same day as questionnaire assessments. All evaluations were conducted during clinical remission, with no acute optic neuritis episodes reported within the preceding 3 months. WHO-defined blindness was defined as best-corrected visual acuity <3/60 (0.05) or a visual field radius ≤10° in the better-seeing eye (21). This binary, severe-impairment classification was used as an objective indicator but does not characterize the full continuum of visual dysfunction.

### 2.3 Vision-related quality of life

VRQoL was assessed using the validated Chinese version of the NEI VFQ-25 (22). The questionnaire evaluates the impact of visual dysfunction across multiple domains, including general vision, near and distance activities, social functioning, mental health, role difficulties, dependency, ocular pain, color vision, and peripheral vision. Scores range from 0 to 100, with higher scores indicating better VRQoL.

The NEI VFQ-25 composite score was analyzed as both a continuous variable and by quartiles. Prespecified domain-specific analyses used conceptually defined scores to summarize visual-function and psychosocial content. Core Visual Function comprised Near Activities, Distance Activities, Peripheral Vision, and Color Vision. Psychosocial content comprised Mental Health, Role Difficulties, Dependency, and Social Functioning. General Vision and Ocular Pain were retained as individual NEI VFQ-25 subscales and were not included in either score because they do not map unambiguously to the two prespecified constructs. These groupings were informed by the content of the instrument and prior psychometric work distinguishing visual-functioning from socioemotional content (12, 13), but were not derived by factor analysis in the present cohort and should not be regarded as validated latent constructs in this population.

### 2.4 Assessment of depressive symptoms

Depressive symptoms were assessed using the depression subscale of the Hospital Anxiety and Depression Scale (HADS-D). The HADS-D comprises seven items, each scored from 0 to 3, yielding a subscale score from 0 to 21 (23). In the present analysis, HADS-D scores ≥11 were retained as the operational threshold for screen positivity and scores ≥15 as a higher symptom-burden threshold for sensitivity analysis. These thresholds characterize questionnaire-based symptom burden and do not establish a psychiatric diagnosis. The ≥11 threshold has been widely used to indicate probable depressive symptom burden; cut-points vary with clinical purpose and population (24, 25).

### 2.5 Statistical analysis

Continuous variables are summarized as mean (standard deviation) or median [interquartile range], as appropriate, and categorical variables as *n* (%). Between-group comparisons used Welch independent-sample *t*-tests, Mann–Whitney *U* tests, chi-square tests, or Fisher’s exact tests, as appropriate. Trend in HADS-D screen-positive prevalence across empirical NEI VFQ-25 quartiles was assessed using the Cochran–Armitage test; HADS-D scores across quartiles were compared using the Kruskal–Wallis test. Comparisons of individual NEI VFQ-25 subscales were exploratory and were interpreted descriptively without adjustment for multiplicity.

Multivariable logistic regression estimated cross-sectional adjusted associations with HADS-D screen positivity (total score ≥11). The full model included NEI VFQ-25 composite score, age, sex, education, marital status, disease duration, disease subtype, WHO-defined blindness, first ocular episode, and history of ocular involvement. NEI VFQ-25 odds ratios (ORs) are expressed per 10-point decrease; age and disease duration are expressed per year and per month, respectively. Education was modeled per category increase (1 = elementary school or below, 2 = junior high, 3 = senior high, and 4 = college or above); marital status was coded as married versus unmarried/divorced/widowed. Sex, disease subtype, and WHO-defined blindness were coded as female versus male, NMOSD versus MS, and yes versus no, respectively. Because HADS-D screen positivity was common in this sample, odds ratios (ORs) are presented as measures of association and should not be interpreted as estimates of relative risk or prevalence ratios.

To further examine the relationship between objective visual impairment, patient-reported visual functioning, and HADS-D screen positivity, additional sequential models were fitted in which WHO-defined blindness and NEI VFQ-25 scores were examined separately and jointly. These analyses were performed to evaluate the extent to which associations with objective visual impairment changed after accounting for vision-related quality of life.

Disease-subtype interaction analyses adjusted for age, sex, education, marital status, and disease duration. Domain-specific and higher-symptom-burden (HADS-D total score ≥15) analyses used this reduced covariate set plus disease subtype; WHO-defined blindness and the two ocular-history variables were not included. Domain analyses examined total and Core Visual Function scores, as well as mutually adjusted Core Visual Function and Psychosocial scores; a sensitivity model used a Psychosocial score excluding the Mental Health subscale. Variance inflation factors were used to assess multicollinearity. Receiver operating characteristic analysis was exploratory and described within-sample apparent discrimination only; the Youden-index threshold was not interpreted as a validated clinical cutoff or prospective prediction.

No missing data were present for variables included in the primary analyses. Linearity of continuous predictors was assessed through inspection of model diagnostics, and no major departures from linearity were identified. Statistical analyses were performed using IBM SPSS Statistics version 26.0 (IBM Corp., Armonk, NY, United States). All tests were two-sided, and *p* < 0.05 was considered statistically significant.

## 3 Results

### 3.1 Participant characteristics

A total of 458 patients were included, comprising 370 patients with MS and 88 with NMOSD. Compared with patients with MS, those with NMOSD were older, more frequently female, and had lower educational attainment. WHO-defined blindness was substantially more prevalent in NMOSD than in MS (27.3% vs. 6.8%, *p* < 0.001). Median HADS-D score was numerically higher in NMOSD than in MS (13.0 vs. 12.0), although this difference did not reach conventional statistical significance (*p* = 0.055); the prevalence of HADS-D screen positivity did not differ significantly between the two groups (70.5% vs. 64.9%, *p* = 0.385). Participant characteristics are summarized in Table 1.

**Table 1:** Demographic, clinical, visual, and depressive symptom characteristics of patients with MS and NMOSD.

	Total (*N* = 458)	MS (*N* = 370)	NMOSD (*N* = 88)	*p*-value
Female sex, *n* (%)	338 (73.8%)	264 (71.4%)	74 (84.1%)	0.021
Age, years	35.0 [29.2, 44.0]	35.0 [29.0, 43.0]	41.5 [32.0, 55.0]	<0.001
Educational level, *n* (%)				0.001†
Elementary school or below, *n* (%)	3 (0.7%)	2 (0.5%)	1 (1.1%)	
Junior high, *n* (%)	73 (15.9%)	53 (14.3%)	20 (22.7%)	
Senior high, *n* (%)	99 (21.6%)	70 (18.9%)	29 (33.0%)	
College and above, *n* (%)	283 (61.8%)	245 (66.2%)	38 (43.2%)	
Marital status, *n* (%)				0.033
Married, *n* (%)	309 (67.5%)	242 (65.4%)	67 (76.1%)	
Unmarried, *n* (%)	115 (25.1%)	103 (27.8%)	12 (13.6%)	
Divorced, *n* (%)	29 (6.3%)	22 (5.9%)	7 (8.0%)	
Widowed, *n* (%)	5 (1.1%)	3 (0.8%)	2 (2.3%)	
Disease duration, months	45.0 [22.0, 88.0]	45.5 [25.0, 87.8]	38.5 [9.8, 109.0]	0.070
First episode with ocular symptoms, *n* (%)	218 (47.6%)	174 (47.0%)	44 (50.0%)	0.702
History of ocular involvement, *n* (%)	223 (48.7%)	175 (47.3%)	48 (54.5%)	0.270
WHO-defined blindness, *n* (%)	49 (10.7%)	25 (6.8%)	24 (27.3%)	<0.001
NEI VFQ-25 composite score (0–100)	88.3 [71.3, 93.4]	88.7 [74.6, 93.7]	78.9 [51.9, 92.0]	<0.001
HADS-D total score	12.0 [9.0, 16.0]	12.0 [9.0, 16.0]	13.0 [10.0, 17.0]	0.055
HADS-D screen positivity, *n* (%)	302 (65.9%)	240 (64.9%)	62 (70.5%)	0.385
Higher symptom-burden threshold (HADS-D ≥ 15), *n* (%)	167 (36.5%)	129 (34.9%)	38 (43.2%)	0.182

Values are median [interquartile range] or *n* (%), as appropriate. Continuous variables were compared using the Mann–Whitney U test. Categorical variables were compared using the chi-square test, continuity-corrected chi-square test, or Fisher–Freeman–Halton exact test, as appropriate. †Exact test for educational level because of sparse cells. WHO-defined blindness was classified according to World Health Organization criteria. HADS-D screen positivity was operationally defined as a total score ≥11; the higher symptom-burden threshold was defined as a total score ≥15. MS, multiple sclerosis; NMOSD, neuromyelitis optica spectrum disorder; HADS-D, Hospital Anxiety and Depression Scale depression subscale; NEI VFQ-25, National Eye Institute Visual Function Questionnaire-25.

### 3.2 Vision-related quality of life across disease groups

VRQoL scores are summarized in Table 2 and Figure 1. Compared with patients with MS, patients with NMOSD reported lower scores in General Vision, Near Activities, Distance Activities, Social Functioning, Mental Health, Role Difficulties, Dependency, Color Vision, and Peripheral Vision (all nominal *p* < 0.05). Ocular Pain did not differ significantly (nominal *p* = 0.146). As these subscale comparisons were exploratory and no multiplicity adjustment was applied, they should be interpreted descriptively.

**Table 2:** Comparison of NEI VFQ-25 subscale scores between patients with MS and NMOSD.

Subscale	Overall (*N* = 458)	MS (*n* = 370)	NMOSD (*n* = 88)	*P*-value*
General vision	52.4 (20.3)	53.7 (19.9)	46.6 (21.2)	0.005
Ocular pain	85.5 (18.6)	86.1 (18.4)	82.8 (19.4)	0.146
Near activities	85.1 (23.6)	87.2 (21.3)	76.3 (30.3)	0.002
Distance activities	84.2 (23.7)	86.3 (20.7)	75.2 (32.1)	0.003
Social functioning	88.9 (22.1)	90.8 (19.9)	80.8 (28.3)	0.002
Mental health	79.4 (27.1)	82.2 (24.9)	67.7 (32.8)	<0.001
Role difficulties	80.0 (28.4)	82.0 (27.2)	71.3 (31.7)	0.004
Dependency	81.5 (28.5)	84.0 (26.2)	70.8 (35.1)	0.001
Color vision	93.8 (16.8)	95.6 (12.9)	86.0 (26.5)	0.002
Peripheral vision	87.3 (21.3)	88.8 (20.2)	80.8 (24.8)	0.007

Values are mean (standard deviation). p values were calculated using two-sided Welch independent-sample t tests. *p values are for MS versus NMOSD. NEI VFQ-25, National Eye Institute Visual Function Questionnaire-25; MS, multiple sclerosis; NMOSD, neuromyelitis optica spectrum disorder.

**Figure 1:**
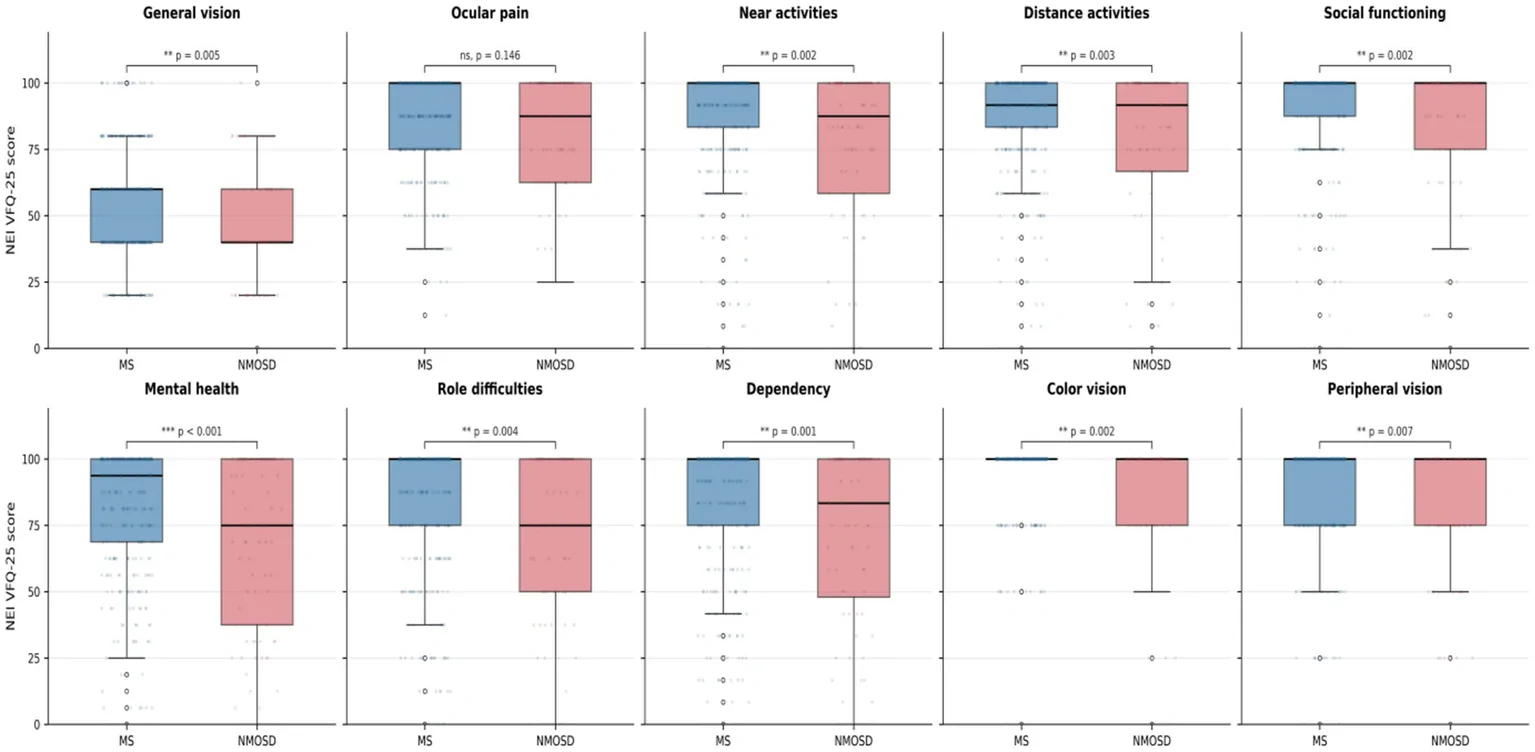
Boxplots show the distribution of NEI VFQ-25 subscale scores in patients with MS and NMOSD. Bars represent mean scores and error bars indicate standard deviations. Mental Health scores were rescaled to the standard 0–100 metric. Between-group comparisons were performed using Welch independent-sample t tests. The individual subscale comparisons were exploratory and were not adjusted for multiple testing. *Nominal *p* < 0.05; **nominal *p* < 0.01; ***nominal *p* < 0.001.

### 3.3 Vision-related quality of life and depressive symptoms

A graded relationship was observed between VRQoL and HADS-D screen positivity (Table 3; Figure 2). The prevalence of screen-positive symptoms (HADS-D total score ≥11) decreased from 90.1% in the lowest NEI VFQ-25 quartile to 38.3% in the highest quartile (*p* for trend <0.001). Median HADS-D scores similarly declined across quartiles, from 16.0 [13.0–19.0] to 9.0 [8.0–12.0] (*p* < 0.001).

**Table 3:** Depressive symptom burden across empirical quartiles of the NEI VFQ-25 composite score.

VFQ-25 quartile	Score range (0–100)	N	Screen-positive symptoms, *n* (%)	HADS-D score, median [IQR]
Q1	3.0–71.3	121	109 (90.1%)	16.0 [13.0–19.0]
Q2	72.2–88.3	111	80 (72.1%)	14.0 [10.0–16.5]
Q3	88.5–93.3	111	69 (62.2%)	11.0 [9.0–14.0]
Q4	93.5–100.0	115	44 (38.3%)	9.0 [8.0–12.0]
*p* value			<0.001†	<0.001‡

† Cochran–Armitage trend test; ‡ Kruskal–Wallis test.

**Figure 2:**
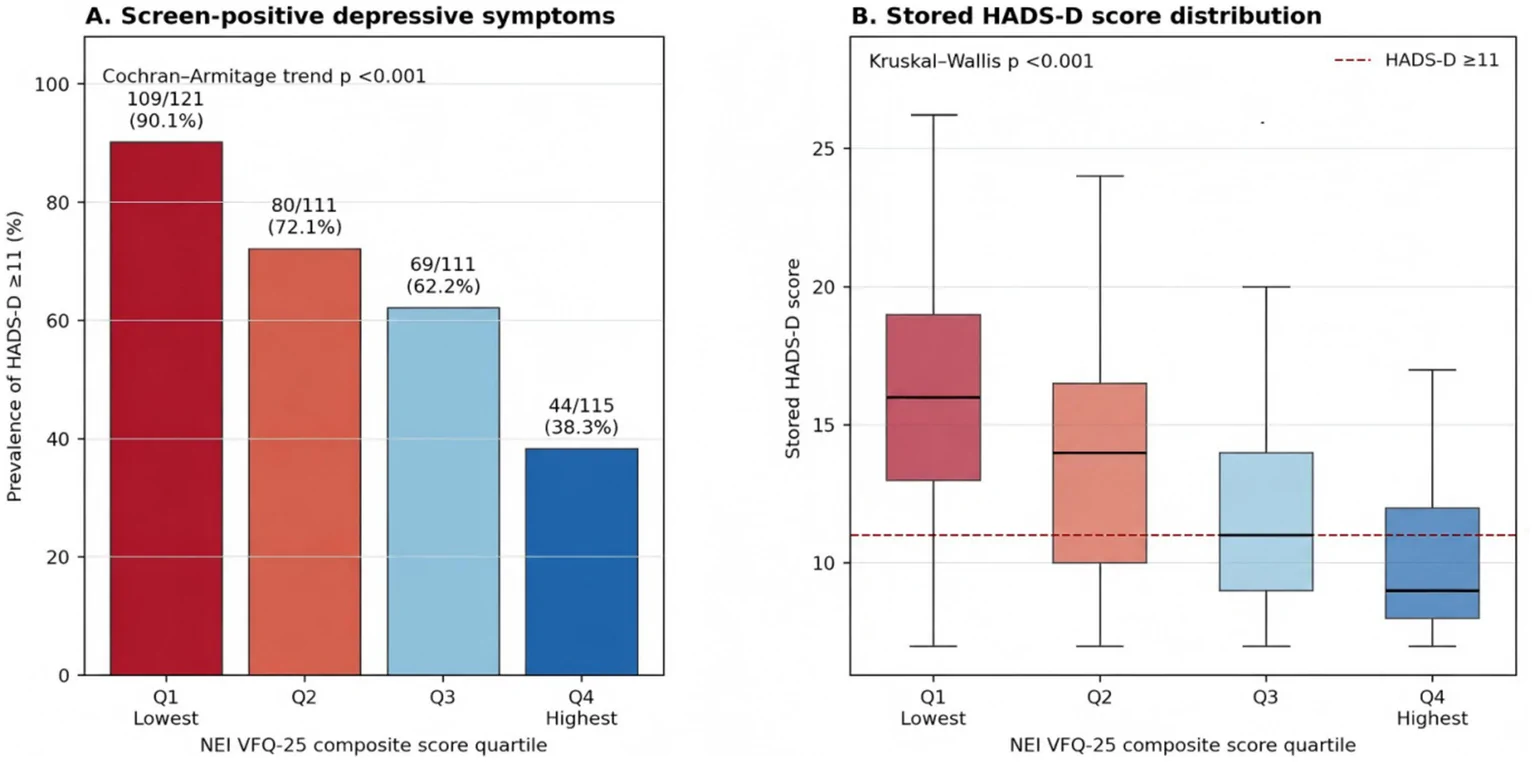
HADS-D screen positivity and score distribution across quartiles of NEI VFQ-25 composite scores. **(A)** Prevalence of HADS-D screen positivity (HADS-D ≥ 11) across empirical quartiles of the NEI VFQ-25. **(B)** Distribution of HADS-D scores across the same quartiles. Central lines indicate medians, boxes indicate interquartile ranges, and whiskers extend to 1.5 × the interquartile range. *p* values were derived from Cochran–Armitage trend and Kruskal–Wallis tests.

Participants were allocated to empirical quartiles of the 0–100 NEI VFQ-25 composite score using the locked analytic dataset. Values are *n* (%) or median [interquartile range]. HADS-D screen positivity was operationally defined as a total score ≥11. *p* values were derived from the Cochran–Armitage trend test for screen-positive symptom prevalence and the Kruskal–Wallis test for HADS-D scores. NEI VFQ-25, National Eye Institute Visual Function Questionnaire-25; HADS-D, Hospital Anxiety and Depression Scale depression subscale.

### 3.4 Adjusted associations between vision-related quality of life and depressive symptoms

In the complete-case multivariable logistic regression model (Figure 3 and Supplementary Table S4), each 10-point decrease in NEI VFQ-25 composite score was associated with higher odds of HADS-D screen positivity after adjustment for the prespecified covariates (OR = 2.33, 95% CI 1.81–3.00; *p* < 0.001). Disease subtype, disease duration, and WHO-defined blindness were not significantly associated with HADS-D screen positivity in the fully adjusted model.

**Figure 3:**
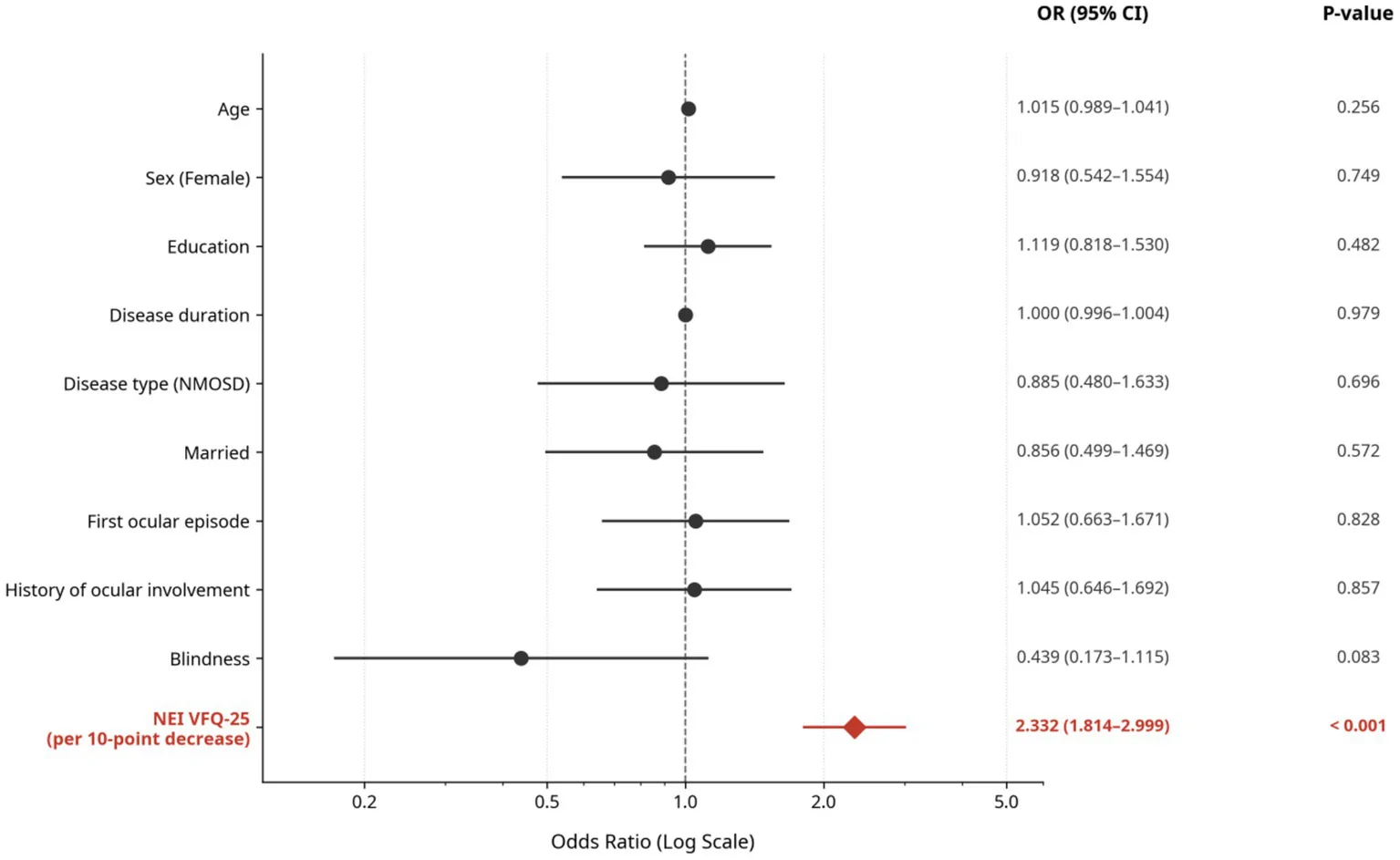
Adjusted associations between vision-related quality of life and HADS-D screen positivity (HADS-D ≥ 11). Forest plot showing adjusted odds ratios (ORs) and 95% confidence intervals (CIs) from the multivariable logistic regression model. NEI VFQ-25 was modeled per 10-point decrease. The reference line indicates OR = 1.

To examine whether the association between objective visual impairment and depressive symptom screening was sensitive to adjustment for patient-reported visual functioning, we fitted three sequential multivariable logistic regression models using the same analytic sample and prespecified covariates. Model A included WHO-defined blindness, Model B included the NEI VFQ-25 composite score, and Model C included both exposures. In Model A, WHO-defined blindness was associated with HADS-D screen positivity (adjusted OR, 2.12; 95% CI, 1.01–4.44; *p* = 0.048). In Model B, a 10-point decrease in NEI VFQ-25 score was associated with higher odds of HADS-D screen positivity (adjusted OR, 1.56; 95% CI, 1.36–1.79; *p* < 0.001). In Model C, the blindness estimate was attenuated and was no longer statistically significant (adjusted OR, 1.21; 95% CI, 0.53–2.75; *p* = 0.652), whereas the association with a 10-point decrease in NEI VFQ-25 remained similar (adjusted OR, 1.55; 95% CI, 1.35–1.78; *p* < 0.001; Supplementary Table S5). These sequential models were used to examine changes in association after joint adjustment and were not interpreted as formal mediation analyses.

In subgroup analyses excluding WHO-defined blindness and ocular-history variables, lower NEI VFQ-25 scores remained associated with HADS-D screen positivity in both MS (OR = 2.14, 95% CI 1.66–2.77; *p* < 0.001) and NMOSD (OR = 1.86, 95% CI 1.24–2.79; *p* = 0.002). No evidence of effect modification by disease subtype was observed (p for interaction = 0.537; Supplementary Table S2).

### 3.5 Relative contributions of visual-function and psychosocial domains

To further explore the relationship between VRQoL and HADS-D screen positivity, the NEI VFQ-25 was summarized as Core Visual Function and Psychosocial content scores (Figure 4 and Supplementary Table S1). These domain-specific models were adjusted for age, sex, educational level, marital status, disease duration, and disease subtype. The scores are conceptually defined summaries rather than empirically derived factors in the present cohort.

**Figure 4:**
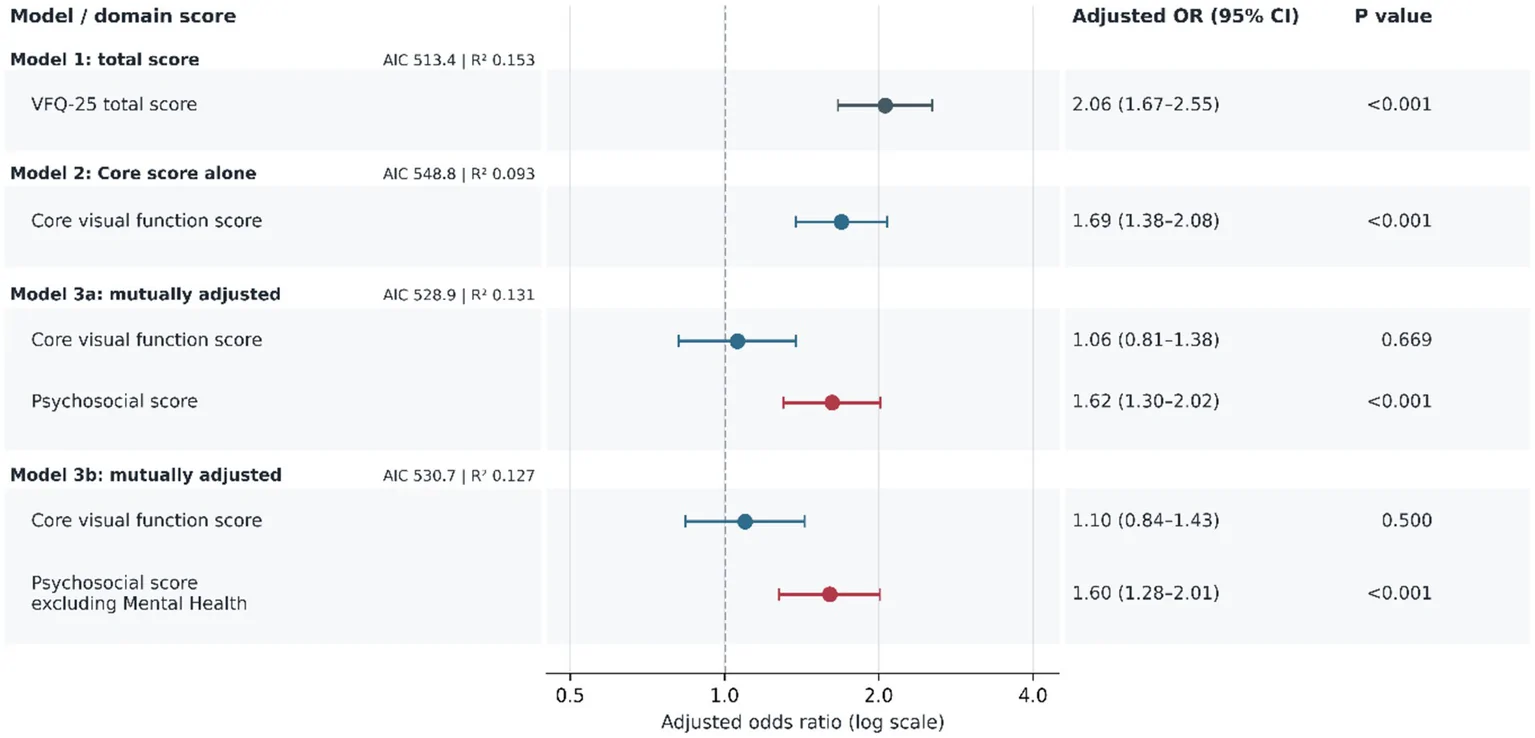
Conceptually defined visual-function and psychosocial content scores in relation to HADS-D screen positivity. Forest plot showing adjusted odds ratios (ORs) and 95% confidence intervals (CIs) per 10-point decrease in NEI VFQ-25 scores. Core Visual Function included Near Activities, Distance Activities, Peripheral Vision, and Color Vision. Psychosocial content included Mental Health, Role Difficulties, Dependency, and Social Functioning. Models were adjusted for age, sex, education, marital status, disease duration, and disease subtype. The scores were conceptually defined and were not derived from factor analysis in the present cohort.

Within the reduced-covariate framework, lower total NEI VFQ-25 scores remained associated with HADS-D screen positivity (OR = 2.06, 95% CI 1.67–2.55; *p* < 0.001; Model 1, Figure 4 and Supplementary Table S1).

When Core Visual Function was entered without a Psychosocial score, each 10-point decrease was associated with higher odds of HADS-D screen positivity (OR = 1.69, 95% CI 1.38–2.08; *p* < 0.001). In mutually adjusted models, the Psychosocial score was associated with HADS-D screen positivity (OR = 1.62, 95% CI 1.30–2.02; *p* < 0.001), whereas the Core Visual Function estimate was attenuated (OR = 1.06, 95% CI 0.81–1.38; *p* = 0.669). In a sensitivity model that excluded the Mental Health subscale from the Psychosocial score, the mutually adjusted estimates were 1.60 (95% CI 1.28–2.01; *p* < 0.001) for the modified Psychosocial score and 1.10 (95% CI 0.84–1.43; *p* = 0.500) for Core Visual Function.

Variance inflation factors were below 3.4, indicating no evidence of substantial multicollinearity. These mutually adjusted cross-sectional models do not establish mediation, temporality, or causal pathways.

### 3.6 Sensitivity analysis using severe depressive symptoms

In sensitivity analyses using the higher HADS-D symptom-burden threshold as the outcome, lower NEI VFQ-25 composite score remained associated with higher odds of symptoms (OR = 1.53, 95% CI 1.35–1.74; *p* < 0.001; Supplementary Table S3).

## 4 Discussion

In this multicenter study of patients with MS and NMOSD, lower vision-related quality of life was associated with HADS-D screen positivity. This association was observed in both diseases and remained evident after adjustment for measured demographic and disease-related variables, including WHO-defined blindness. Although the Psychosocial score was associated with HADS-D screen positivity in mutually adjusted models and the Core Visual Function estimate was attenuated, the cross-sectional design does not permit conclusions about temporal relationships, mediation, or underlying mechanisms. Taken together, the findings indicate that patient-reported visual dysfunction captures aspects of disease burden that may not be fully reflected by a binary classification of blindness.

A notable finding was that lower vision-related quality of life remained associated with HADS-D screen positivity, whereas WHO-defined blindness was not independently associated in the fully adjusted model. Objective measures of visual impairment remain essential components of neuro-ophthalmic assessment, but a binary blindness classification does not capture the full spectrum of visual dysfunction, daily functioning, or vision-related quality of life (2, 3, 7). The present data should not be interpreted as showing that patient-reported outcomes are independent of underlying visual impairment; objective impairment can contribute to patient-reported visual difficulties. Rather, validated patient-reported measures may complement clinical examination and testing by capturing patients’ perceived functional impact (9, 10, 26).

The domain-specific analyses provided additional detail on the observed cross-sectional associations. Lower Core Visual Function scores were associated with HADS-D screen positivity when the Psychosocial score was not included; this estimate was attenuated in mutually adjusted models. The Psychosocial score remained associated with HADS-D screen positivity, including in the sensitivity model excluding Mental Health. These results should be interpreted cautiously. The conceptually defined questionnaire scores may overlap with broader emotional and functional well-being, and the analysis was not designed as a formal mediation analysis. The results therefore do not show that psychosocial consequences of visual dysfunction cause depressive symptoms; they describe associations within this cross-sectional dataset.

Visual dysfunction remains an important source of disability in both MS and NMOSD, even in the absence of complete blindness. Many patients experience difficulties with reading, mobility, employment, and social participation following optic neuritis or other forms of visual pathway involvement (2, 6, 21). Such difficulties may persist despite stabilization of visual acuity and contribute to perceived disease burden. The present findings are consistent with previous evidence that visual difficulties, quality of life, and psychological symptoms are related in people with inflammatory demyelinating disease (5, 17, 27, 28).

The clinical implications of these findings should be interpreted cautiously. Assessment of patient-reported visual difficulties may help identify individuals whose functional and psychosocial burden is not readily apparent from ophthalmological examination alone. Validated patient-reported outcome measures may complement, rather than replace, clinical examination and objective visual testing in routine MS care (9, 10). Qualitative evidence from MS care further suggests that patients value systematically collected PROMs when clinicians review the results, although the choice and implementation of PROMs should remain individualized (9). In NMOSD, this broader approach is also consistent with recent calls to treat mental health as an unmet clinical need alongside attention to relapse fear, stigma, diagnostic burden, and caregiver impact (29).

This study has several strengths. The multicenter design and inclusion of patients from both MS and NMOSD cohorts provide a broad clinical context for the findings. The study population encompassed a range of reported visual-function scores and WHO-defined blindness status. Finally, the direction of the association between lower NEI VFQ-25 composite score and HADS-D screen positivity was consistent across the primary, subgroup, and higher-threshold analyses.

This study has several limitations. First, the cross-sectional design precludes conclusions regarding temporal or causal relationships. Second, WHO-defined blindness is an extreme binary measure and does not capture the full spectrum of visual dysfunction in MS and NMOSD, including low-contrast visual deficits and retinal structural changes detectable by optical coherence tomography (30, 31). Third, both the NEI VFQ-25 and HADS-D are self-reported measures and may be influenced by shared reporting tendencies, negative affectivity, and common-method bias (29). The conceptually defined domain scores do not eliminate potential overlap between visual, functional, and emotional constructs and should not be interpreted as formal mediators. Fourth, HADS-D is a screening instrument rather than a structured psychiatric interview; the observed screen-positive prevalence may also reflect recruitment from tertiary referral centers and the medication-related exclusion criterion. Fifth, recruiting-centre effects and clustering were not evaluated in the primary model and may have influenced the estimates. Finally, standardized EDSS scores were unavailable. Residual confounding by neurological disability, fatigue, pain, cognition, disease activity, treatment status, and other disease-related factors cannot be excluded. Future longitudinal studies incorporating standardized neuro-ophthalmic, neurological, treatment, and psychiatric assessments are needed.

## 5 Conclusion

In patients with multiple sclerosis and neuromyelitis optica spectrum disorder, lower vision-related quality of life was associated with HADS-D screen positivity. In mutually adjusted models, the Psychosocial score remained associated with HADS-D screen positivity, whereas the Core Visual Function estimate was attenuated. These conceptually defined, cross-sectional analyses do not identify a causal pathway. Vision-related patient-reported outcomes may provide clinically useful information about disease burden that is not fully reflected by a binary classification of visual impairment.

## Data Availability

The original contributions presented in the study are included in the article/Supplementary material, further inquiries can be directed to the corresponding authors.
