# Supplementary material for: Association between psychosocial consequences of visual dysfunction and depressive symptom burden in multiple sclerosis and neuromyelitis optica spectrum disorder
Source: Front Neurol. 2026 Oct 20;17:1975600. doi: 10.3389/fneur.2026.1975600 (PMC13727409; doi:10.3389/fneur.2026.1975600)
Supplement: Supplementary file 1 [file Supplementary_file_1.docx]

**Title: Association between psychosocial consequences of visual dysfunction and depressive symptom burden in multiple sclerosis and neuromyelitis optica spectrum disorder**

**Supplementary Materials**

**Table S1. Relative contributions of visual-function and psychosocial domains to HADS-D screen positivity**

| **Model** | **Predictor** | **OR (95% CI)** | **P-value** | **AIC** | **McFadden pseudo-R²** |
| --- | --- | --- | --- | --- | --- |
| Model 1 | VFQ-25 Total Score | 2.06 (1.67–2.55) | <0.001 | 513.4 | 0.153 |
| Model 2 | Core Visual Function Score | 1.69 (1.38–2.08) | <0.001 | 548.8 | 0.093 |
| Model 3a | Core Visual Function Score | 1.06 (0.81–1.38) | 0.669 | 528.9 | 0.131 |
| Model 3a | Psychosocial Score | 1.62 (1.30–2.02) | <0.001 | 528.9 | 0.131 |
| Model 3b | Core Visual Function Score | 1.10 (0.84–1.43) | 0.500 | 530.7 | 0.127 |
| Model 3b | Modified Psychosocial Score (excluding Mental Health) | 1.60 (1.28–2.01) | <0.001 | 530.7 | 0.127 |

**Outcome:** **HADS-D screen positivity (HADS-D ≥11)**. For all NEI VFQ-25 predictors, odds ratios (ORs) are expressed per 10-point decrease. Domain-specific models were adjusted for age, sex, educational level, marital status, disease duration, and disease subtype. WHO-defined blindness, first ocular episode, and history of ocular involvement were not included in these reduced-covariate models. Core Visual Function was defined as the mean of the Near Activities, Distance Activities, Peripheral Vision, and Color Vision subscales. Psychosocial Score was defined as the mean of the Mental Health, Role Difficulties, Dependency, and Social Functioning subscales. The modified Psychosocial Score excluded the Mental Health subscale. Variance inflation factors in mutually adjusted models were below 3.4, indicating no evidence of substantial multicollinearity. These domain groupings were conceptually prespecified to examine the relative contributions of visual-function and psychosocial components of vision-related quality of life and were not derived from factor analysis in the present cohort.

**Table S2. Disease-subgroup analyses of associations between NEI VFQ-25 score and HADS-D screen positivity**

| Subgroup | N | Screen-positive n (%) | OR per 10-point decrease (95% CI) | P-value | P for interaction |
| --- | --- | --- | --- | --- | --- |
| MS | 370 | 240 (64.9%) | 2.14 (1.66–2.77) | <0.001 | 0.537 |
| NMOSD | 88 | 62 (70.5%) | 1.86 (1.24–2.79) | 0.002 |  |

**Outcome: HADS-D screen positivity (HADS-D ≥11).** NEI VFQ-25 ORs are expressed per 10-point decrease. Subgroup models were adjusted for age, sex, education level (per category increase), marital status (married vs unmarried/divorced/widowed), and disease duration. The interaction p value was obtained from a model containing NEI VFQ-25 score, disease type, and their product term, with the same covariates. WHO-defined blindness, first ocular episode, and history of ocular involvement were excluded from this reduced covariate set. No evidence of effect modification by disease subtype was observed.

**Table S3. Sensitivity analysis using severe depressive symptoms as the outcome**

| **Predictor** | **OR per 10-point decrease (95% CI)** | **P-value** |
| --- | --- | --- |
| VFQ-25 Total Score | 1.53 (1.35–1.74) | <0.001 |
| Core Visual Function Score | 1.39 (1.23–1.57) | <0.001 |
| Modified Psychosocial Score (excluding Mental Health) | 1.36 (1.23–1.50) | <0.001 |

**Outcome: Higher symptom-burden threshold (HADS-D ≥15).** For all NEI VFQ-25 predictors, ORs are expressed per 10-point decrease. Models were adjusted for age, sex, educational level (modeled per category increase), marital status, disease duration, and disease subtype. WHO-defined blindness, first ocular episode, and history of ocular involvement were excluded from the reduced-covariate model used for sensitivity analyses.

**Table S4. Complete-case multivariable logistic regression model for HADS-D screen positivity**

| **Variable** | **OR** | **95% CI** | **P value** |
| --- | --- | --- | --- |
| Age (per year) | 1.015 | 0.989–1.041 | 0.256 |
| Female sex (vs male) | 0.918 | 0.542–1.554 | 0.749 |
| Education level (per category increase) | 1.119 | 0.818–1.530 | 0.482 |
| Disease duration (per month) | 1.000 | 0.996–1.004 | 0.979 |
| NMOSD (vs MS) | 0.885 | 0.480–1.633 | 0.696 |
| Married (vs unmarried/divorced/widowed) | 0.856 | 0.499–1.469 | 0.572 |
| First ocular episode (yes vs no) | 1.052 | 0.663–1.671 | 0.828 |
| History of ocular involvement (yes vs no) | 1.045 | 0.646–1.692 | 0.857 |
| WHO-defined blindness (yes vs no) | 0.439 | 0.173–1.115 | 0.083 |
| NEI VFQ-25 composite score (per 10-point decrease) | 2.332 | 1.814–2.999 | <0.001 |

**Outcome:** **HADS-D screen positivity (HADS-D ≥11).** All listed variables were entered simultaneously. Age and disease duration were modeled continuously and are expressed per year and per month, respectively. Educational level was coded from 1 (elementary school or below) to 4 (college or above) and modeled per category increase. NEI VFQ-25 odds ratios are expressed per 10-point decrease. In the complete-case model, variance inflation factors ranged from 1.08 to 1.59.

**Table S5. Sequential associations of WHO-defined blindness and NEI VFQ-25 score with HADS-D screen positivity**

| **Model** | **Exposure** | **Adjusted OR (95% CI)** | **P value** |
| --- | --- | --- | --- |
| A | WHO-defined blindness only | 2.12 (1.01–4.44) | 0.048 |
| B | NEI VFQ-25, per 10-point decrease | 1.56 (1.36–1.79) | <0.001 |
| C | WHO-defined blindness | 1.21 (0.53–2.75) | 0.652 |
| C | NEI VFQ-25, per 10-point decrease | 1.55 (1.35–1.78) | <0.001 |

**Outcome:** **HADS-D screen positivity (HADS-D ≥11).** All models used the same analytic sample and the same prespecified covariates: age, sex, education, marital status, disease duration, disease subtype, first ocular episode, and history of ocular involvement. Model A included WHO-defined blindness, Model B included the NEI VFQ-25 composite score, and Model C included both exposures. NEI VFQ-25 was modeled per 10-point decrease. Changes in the blindness estimate were interpreted as attenuation after joint adjustment and not as evidence of mediation or causal pathways.
